# Distribution of HPV genotypes and comparison of cervico-vaginal cytologies in PCR-detected HPV-positive patients: a retrospective observational study

**DOI:** 10.3389/fgwh.2026.1780272

**Published:** 2026-02-24

**Authors:** Mehmet Nuri Duran, Serem Kel Ilgin, Süreyya Saridas Demir, Bülent Demir, Fatma Silan

**Affiliations:** 1 Department of Obstetrics and Gynecology, Medical Faculty of Canakkale Onsekiz Mart University, Canakkale, Türkiye; 2 Department of Obstetrics and Gynecology, Biga State Hospital, Canakkale, Türkiye; 3 Department of Perinatology, Clinics, Canakkale, Türkiye; 4 Department of Genetics, Faculty of Medicine, Çanakkale Onsekiz Mart University, Çanakkale, Türkiye

**Keywords:** cervical cancer, cervical cytology, HPV genotypes, HPV-16, human papillomavirus

## Abstract

**Background:**

This large-scale study aimed to determine the prevalence and genotype distribution of Human Papillomavirus (HPV) infection together with cervical cytological findings among women undergoing cervical screening in Türkiye. In Türkiye, HPV prevalence varies regionally, and vaccination rates remain low due to the absence of HPV vaccination in the national program.

**Methods:**

A retrospective study of 4,648 women who underwent HPV testing and cervical cytology from January 2020 to March 2023. HPV genotype was determined by multiple PCR. Cytological findings were categorized based on the 2015 Bethesda System. Statistical analysis was conducted using the chi-square test and logistic regression to compare the association between positivity for HPV, age groups, types of infection, and cytological alterations.

**Results:**

17.7% (*n* = 825) of both groups were HPV positive, and 82.3% of the subjects (*n* = 3,823) were negative for HPV. HPV positivity had a bimodal distribution by age, with higher prevalence among women <30 years and those aged 40–49 (*p* = 0.0049). Women younger than 29 years had significantly higher odds of being HPV positive compared with the reference group (OR: 2.55; 95% CI: 1.86–3.49; *p* < 0.01). The most prevalent genotypes were HPV-16 (23.3%) followed by HPV-6 (13.3%), HPV-51 (6.3%), and HPV-59 (6.5%). Differences in cytological distributions between single and multiple infections were not significant (*p* = 0.41), though HPV-16 was significantly associated with moderate-severe lesions (HSIL, ICC) (*p* < 0.01).

**Conclusion:**

The results indicate the requirement for age-optimized screening, ongoing genotype surveillance, and successful HPV vaccination programs. Cervical cancer represents a major health burden although it is largely preventable through vaccination. These findings highlight the urgent need for genotype-specific surveillance and reinforce the importance of implementing comprehensive HPV vaccination programs in Türkiye.

## 1 Introduction

Human papillomavirus infection is the known cause of more than 5% of all malignant diseases associated with infections and includes cervical, vaginal, vulvar, anal, and oropharyngeal cancer, as well as one of the major causes of cervical cancer, which remains one of the top chronic diseases globally for women's health. It's also the most common viral sexually transmitted infection worldwide (1, 2). Cervical cancer ranks as the fourth most common malignancy in women and is among the leading causes of cancer-related deaths worldwide, particularly in low- and middle-income countries (3). However, screening for cervical cancer can identify precancerous changes (cervical dysplasia) and early-stage cervical cancers. Persistent infection with high-risk human papillomavirus (HR-HPV) genotypes is recognized as the necessary etiological factor for cervical carcinogenesis, with HPV-16 and HPV-18 responsible for approximately 70% of cases worldwide (4). HPV prevalence varies from country to country based on the rates of vaccination and screening. However, geographic heterogeneity of genotype distribution is remarkable, and knowledge of these variations is indispensable to tailor screening and prevention programs. These results suggest that local and global genotype landscapes often diverge from each other, which raises the issue of region-specific vaccination strategies. In addition, modeling estimates that increasing vaccine coverage, particularly in areas with low access, could avert hundreds of thousands of additional cases of cervical cancer each year (5).

In Türkiye, HPV prevalence exhibits substantial variability across centers, with reported rates ranging from about 10% to 40% (6, 7). Because the HPV vaccine has not yet been included in the national immunization program, vaccination uptake remains very low, with published rates ranging from 1.4% to 3.6% (8, 9). High-risk HPV prevalence may reach 18%–24% in some cohorts, and HPV-16 consistently remains the predominant genotype nationwide (7, 10). In addition to cervical cancer, HPV is associated with a range of cancers that affect both men and women, such as anal, vulvar, vaginal, penile, and oropharyngeal. Its role is different among anatomical sites, though. For example, an analysis of studies on oral cavity squamous cell carcinoma (primary site, oral cavity and oropharynx) found that HPV was present in just 6% of tumors, indicating a substantially less strong etiologic relationship than for oropharyngeal cancer (11). These subtleties reinforce the need to interpret genotype prevalence and oncogenic potential in an anatomic and epidemiologic perspective. The landscape of prevention has been dramatically altered with the introduction of HPV vaccination. Results of both clinical and population-based trials support strong declines in the prevalence of HPV-16 and HPV-18 after vaccine introduction.

Nonetheless, the threat of genotype replacement or persistence by non-vaccine types such as HPV-52, HPV-58, and HPV-31, which continue to cause high-grade lesions in vaccinated populations, exists (12, 13). Worldwide investigations demonstrated that currently available vaccines include most, but not all, of the genotypes responsible for cancer development, and highlight the significance of continued monitoring as well as improving vaccine versions (14). Screening is just as important in the age of a vaccine. Conventional Pap cytology, although very effective, is less sensitive than HPV testing. Co-testing and primary HPV testing have become more popular, reported to increase the detection rate of high-grade intraepithelial lesions (15, 16). Moreover, population-based programs in Europe and Asia have shown that adding HPV testing to national screening strategies leads to earlier detection and a lower incidence of invasive cancers (16). Therefore, the description of the distribution of HPV genotypes has become even more vital for further knowledge on the epidemiology and aetiological aspects of the disease for health preventive strategies.

## 2 Methods

### 2.1 Study design and population

This retrospective observational study was conducted at the Çanakkale Onsekiz Mart University Faculty of Medicine, Department of Obstetrics and Gynecology. The study was approved by the Ethics Committee of the same university. The study was conducted in accordance with the principles of the Declaration of Helsinki. Demographic, clinical, and laboratory information of the participants was retrieved from the hospital's electronic database, which includes standardized records routinely collected during gynecological visits. The study population consisted of women attending the outpatient gynecology clinic for routine examinations, cervical cancer screening, or various gynecologic complaints. Between January 2020 and March 2023, 4,648 women were included. Among them, 3,823 were HPV negative and 825 were HPV positive. Among these, 825 women were identified as HPV positive following molecular testing. Cervical smear specimens were collected during routine gynecological examinations using a sterile cytobrush. The samples were immediately fixed in 95% alcohol and processed according to standard cytological protocols. Slides were stained and examined by experienced cytopathologists under a light microscope. Cytological interpretations were reported according to the 2015 Bethesda System as: Negative for intraepithelial lesion or malignancy (NILM), atypical squamous cells of undetermined significance (ASCUS), low-grade squamous intraepithelial lesion (LSIL), high-grade squamous intraepithelial lesion (HSIL), invasive squamous carcinoma, and inadequate samples (17). This standardized classification ensured consistency in reporting and comparability with international studies. The 50–65 years age group was selected as the reference category because it represented the highest age range available for comparison and demonstrated the lowest HPV positivity rate in the cohort. In addition, routine HPV testing is not recommended or reimbursed for individuals over 65 years of age according to national screening policies.

### 2.2 Data collection

Sociodemographic characteristics (including age and parity), clinical information, and cervical cytology results were obtained from institutional electronic medical records. HPV testing was performed as part of routine gynecological care. All sample collections, cytological evaluations, and HPV genotyping procedures were performed in our tertiary university hospital by the same institutional clinical and laboratory team.

### 2.3 Sample collection and laboratory analysis

Cervical exfoliated cells were collected using a cytobrush during speculum examination and preserved in a liquid-based cytology medium. Genomic DNA was extracted from cervicovaginal smear specimens with the QIAamp Blood and Tissue Kit (QIAGEN, Hilden, Germany), following the manufacturer's guidelines. HPV DNA detection and genotyping were performed using the F-HPV typing™ multiplex PCR kit (Molgentix SL, Barcelona, Spain). This assay detects HPV genotypes individually, and it provides the direct, type-specific identification of each HPV genotype. All sampling procedures and laboratory analyses were performed in our tertiary university hospital by the same institutional clinical and laboratory team, and the results were subsequently recorded in the hospital electronic database.

### 2.4 Inclusion and exclusion criteria

**Figure 1:**
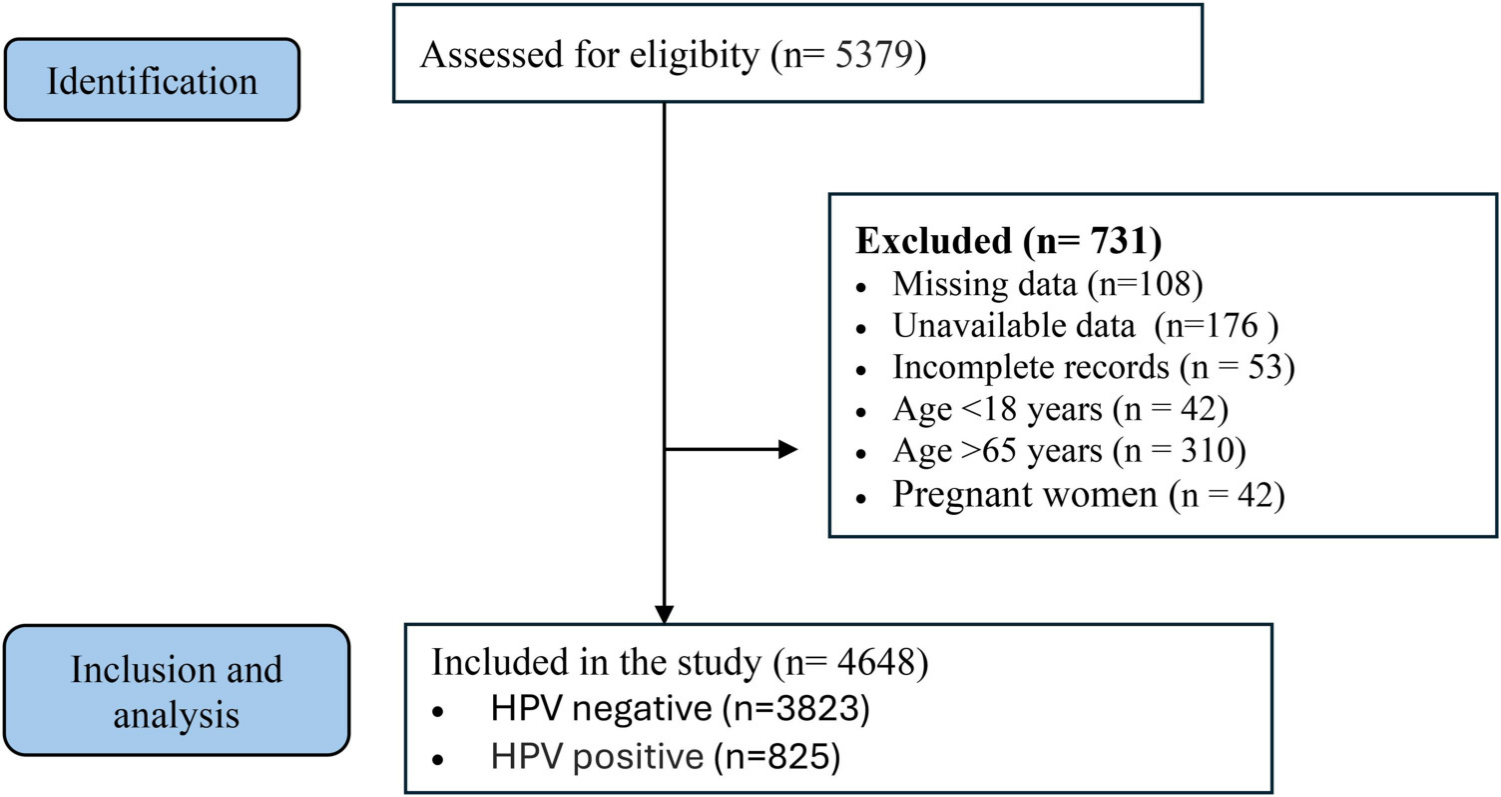
Strengthening the reporting of observational studies in epidemiology (STROBE). Flow diagram of patients recruitment.

Inclusion criteria were defined as women who underwent routine gynecological examination with both HPV testing and cervical smear sampling, had no history of gynecologic malignancy, had an intact uterus and cervix, and whose demographic and laboratory data were fully accessible for evaluation. Exclusion criteria were defined as women younger than 18 or older than 65 years, pregnant women, those with a history of hysterectomy or cervical conization, those with uterine prolapse, patients with incomplete demographic or clinical records, and cases in which HPV test results were unavailable or specimens were inadequate for analysis (Figure 1).

### 2.5 Statistical analysis

All statistical analyses were performed using SPSS version 26 (IBM Corp., Armonk, NY, USA). Categorical variables were presented as frequencies and percentages. Differences in HPV positivity across age groups, genotype distribution, infection type (single vs. multiple), and cytology categories were evaluated using the chi-square (*χ*^2^) test; when significant, *post-hoc* analyses based on adjusted residuals were applied to identify the source of significance. To examine the relationship between HPV positivity and age groups, as well as the association between infection type and cytological abnormalities, logistic regression analyses were conducted. Results were expressed as odds ratios (ORs) with 95% confidence intervals (CIs). Additional regression models were constructed to assess the association between high-grade lesions (HSIL/ICC) and specific HPV genotypes, particularly HPV-16. A two-tailed *p*-value < 0.05 was considered statistically significant for all analyses.

## 3 Results

A total of 4,648 women were included in the study. Of these, 82.3% (*n* = 3,823) were HPV negative and 17.7% (*n* = 825) were HPV positive. Logistic regression analysis was conducted to examine how HPV positivity varied across age groups. Using women aged 50–65 years as the reference category, the model showed that age was an important predictor of HPV positivity (*p* < 0.01). Women younger than 30 years had significantly higher odds of being HPV positive compared with the reference group (OR: 2.55; *p* < 0.01). A similar pattern was observed in the 40–49 year age group, which also demonstrated an increased risk (OR: 1.77; *p* < 0.01). In contrast, women aged 30–39 years had only a slight, non-significant rise in HPV positivity (OR: 1.32; *p* = 0.08). Overall, the findings indicate a clear bimodal distribution, with HPV positivity peaking in women under 30 years of age and again in those aged 40–49 years. This regression pattern confirms that these two age groups carry considerably higher risk of HPV infection compared with women aged 50–65 years (Table 1).

**Table 1:** Logistic regression analysis of the association between age groups and HPV positivity.

Age group	HPV test negative n(%)	HPV test positive n(%)	OR	95% CI	Total n	*p*-value
<30	72.7% (758)	27.3% (285)	2.55	1.86–3.49	1,043	<0.001
30–39	83.7% (1,139)	16.3% (231)	1.32	0.96–1.82	1,361	0.086
40–49	79.1% (895)	20.9% (236)	1.77	1.27–2.47	1,131	0.001
50–65	87% (488)	13% (73)	1.00	—	561	—
Total	82.3% (3,823)	17.7% (825)			4,648	

Binary logistic regression model using HPV positivity as the dependent variable and age group as the predictor (reference: 50–65 years). OR values >1 indicate higher odds of HPV positivity. CI, Confidence Interval; OR, Odds ratio.

When the relationship between age groups and infection type (single vs. multiple) was examined, the distribution of infection types was found to be unequal. The proportion of multiple infections was higher than expected in the <30 year group. Multiple infections were particularly more common in the younger age group (<30 years), which was statistically significant (OR: 1.46; *p* = 0.01). In contrast, the 40–49 year group showed a lower-than-expected proportion of multiple infections (*p* < 0.001) (Table 2).

**Table 2:** HPV genotypes distribution among 825 HPV positive cases by age groups.

HPV genotype	≤29 years	30–39 years	40–49 years	50–65 years	Total	*p*
Single type	21.8% (180)	19.4% (160)	22.5% (186)	5.0% (41)	68.7% (567)	<0.001
Multiple type	12.7% (105)	8.6% (71)	6.1% (50)	3.9% (32)	31.3% (258)	
Total	34.5% (285)	28.0% (231)	28.6% (236)	8.8% (73)	100.0% (825)	
HPV-6	5.5% (45)	3.4% (28)	3.5% (29)	1.0% (8)	13.3% (110)	
HPV-11	1.0% (8)	0.7% (6)	0.7% (6)	0.1% (1)	2.5% (21)	
HPV-16	7.6% (63)	7.4% (61)	6.1% (50)	2.2% (18)	23.3% (192)	
HPV-18	1.8% (15)	1.3% (11)	1.7% (14)	0.5% (4)	5.3% (44)	
HPV-31	1.0% (8)	1.0% (8)	0.5% (4)	0.7% (4)	2.9% (24)	
HPV-33	0.9% (8)	0.7% (6)	0.6% (5)	0.0% (0)	2.3% (19)	
HPV-35	0.6% (5)	0.5% (4)	0.3% (3)	0.1% (1)	1.6% (13)	
HPV-39	1.8% (15)	1.3% (11)	1.6% (13)	0.4% (3)	5.1% (42)	
HPV-45	1.1% (9)	0.5% (4)	0.5% (4)	0.5% (4)	2.5% (21)	
HPV-51	2.1% (17)	1.7% (14)	1.7% (14)	0.8% (7)	6.3% (52)	
HPV-52	1.6% (13)	1.6% (13)	1.9% (16)	0.4% (3)	5.2% (43)	
HPV-56	0.8% (7)	1.7% (14)	2.3% (19)	0.4% (3)	5.2% (43)	
HPV-58	1.0% (8)	0.7% (6)	1.3% (11)	0.5% (4)	3.5% (29)	
HPV-59	2.5% (21)	1.6% (13)	1.9% (16)	0.5% (4)	6.5% (54)	
HPV-66	1.8% (15)	1.0% (8)	1.3% (11)	0.6% (5)	4.7% (39)	
Other genotypes	3.4% (28)	2.9% (24)	2.5% (21)	0.7% (6)	9.6% (79)	

The *p*-value was obtained using the chi-square test.

No significant differences were observed in the cytology distribution between single and multiple HPV infections (*p* = 0.41). However, the cytology distribution of HPV-16 positive cases differed significantly compared with other HPV types (*p* < 0.001). HPV-16 was more frequently detected in high-grade lesions (HSIL, ICC). The most frequently identified genotype was HPV-16 (23%), followed by HPV-6 (13.3%), HPV-51 (6.6%), and HPV-59 (6.2%). Logistic regression analysis revealed that HPV-16 was the only genotype significantly associated with an increased risk of HSIL/ICC (OR: 6.40; *p* < 0.001). None of the other genotypes—including HPV-18, HPV-31, HPV-33, HPV-52, and HPV-66—showed a meaningful association with high-grade lesions. Similarly, multiple infections did not confer additional risk compared with single infections (Table 3).

**Table 3:** Distribution of HPV genotypes according to cervical cytology and logistic regression analysis for HSIL/ICC risk.

HPV	ASCUS	CIN 1	CIN 2	CIN 3	HSIL	ISC	LSIL	Normal	Inadequate sample	Total	OR (HSIL/ICC Risk)	*p*-value
Single type	19 (3.7%)	4 (0.6%)	0 (0%)	1 (0.2%)	6 (1.1%)	1 (0.2%)	18 (2.9%)	514 (90.7%)	4 (1.1%)	567 (100%)	1.00 (ref)	-
Multiple type	11 (4.2%)	2 (0.8%)	1 (0.4%)	0 (0%)	3 (1.1%)	0 (0%)	13 (5.0%)	229 (87.7%)	1 (0.4%)	258 (100%)	1.21	0.41
Group 1												
HPV 6	3 (2.7%)	0 (0.0%)	1 (0.9%)	0 (0.0%)	1 (0.9%)	1 (0.9%)	2 (1.8%)	102 (92.7%)	0 (0.0%)	110 (100.0%)	-	-
HPV-11	0 (0.0%)	1 (4.8%)	0 (0.0%)	0 (0.0%)	1 (4.8%)	0 (0.0%)	2 (9.5%)	17 (80.9%)	0 (0.0%)	21 (100.0%)	-	-
HPV-16	9 (3.9%)	2 (0.8%)	0 (0.0%)	0 (0.0%)	7 (2.7%)	1 (0.4%)	16 (6.2%)	157 (81.8%)	0 (0.0%)	192 (100.0%)	6.40	<0.001
HPV-18	1 (1.6%)	2 (3.2%)	1 (1.6%)	1 (1.6%)	0 (0.0%)	0 (0.0%)	1 (1.6%)	38 (86.3%)	0 (0.0%)	44 (100.0%)	1.85	0.25
HPV-31	1 (2.8%)	0 (0.0%)	0 (0.0%)	0 (0.0%)	1 (2.8%)	0 (0.0%)	4 (11.1%)	18 (75.0%)	0 (2.8%)	24 (100.0%)	1.42	0.64
HPV-33	2 (5.4%)	0 (0.0%)	0 (0.0%)	0 (0.0%)	0 (0.0%)	0 (0.0%)	3 (8.1%)	14 (73.3%)	0 (0.0%)	19 (100.0%)	1.10	0.92
HPV-35	2 (9.1%)	0 (0.0%)	0 (0.0%)	0 (0.0%)	0 (0.0%)	0 (0.0%)	1 (4.5%)	10 (76.9%)	0 (0.0%)	13 (100.0%)	1.20	0.85
HPV-39	5 (7.8%)	1 (1.6%)	0 (0.0%)	0 (0.0%)	0 (0.0%)	0 (0.0%)	2 (3.1%)	34 (81.0%)	0 (0.0%)	42 (100.0%)	0.65	0.69
HPV-45	1 (4.3%)	0 (0.0%)	0 (0.0%)	0 (0.0%)	0 (0.0%)	0 (0.0%)	0 (0.0%)	20 (95.2%)	0 (0.0%)	21 (100.0%)	-	-
HPV-51	2 (3.8%)	0 (0.0%)	0 (0.0%)	0 (0.0%)	0 (0.0%)	0 (0.0%)	4 (7.1%)	45 (86.5%)	1 (1.9%)	52 (100.0%)	1.04	0.96
HPV-52	2 (4.2%)	0 (0.0%)	0 (0.0%)	0 (0.0%)	1 (2.1%)	0 (0.0%)	2 (4.7%)	40 (93.0%)	0 (0.0%)	43 (100.0%)	2.05	0.47
HPV-56	1 (2.3%)	0 (0.0%)	0 (0.0%)	0 (0.0%)	0 (0.0%)	0 (0.0%)	1 (2.3%)	41 (95.3%)	0 (0.0%)	43 (100.0%)	-	-
HPV-58	2 (7.2%)	0 (0.0%)	1 (3.4%)	0 (0.0%)	0 (0.0%)	0 (0.0%)	2 (7.2%)	24 (82.8%)	0 (0.0%)	29 (100.0%)	1.85	0.50
HPV-59	1 (1.9%)	0 (0.0%)	0 (0.0%)	0 (0.0%)	0 (0.0%)	0 (0.0%)	2 (3.8%)	50 (92.6%)	1 (1.9%)	54 (100.0%)	1.02	0.98
Group 2A												
HPV-68	1 (6.2%)	0 (0.0%)	0 (0.0%)	0 (0.0%)	0 (0.0%)	0 (0.0%)	0 (0.0%)	15 (93.8%)	0 (0.0%)	16 (100.0%)	-	-
Group 2B												
HPV-53	0 (0.0%)	0 (0.0%)	0 (0.0%)	0 (0.0%)	0 (0.0%)	0 (0.0%)	0 (0.0%)	4 (100.0%)	0 (0.0%)	4 (100.0%)	-	-
HPV-66	1 (1.8%)	0 (0.0%)	1 (1.8%)	0 (0.0%)	1 (1.8%)	0 (0.0%)	0 (0.0%)	35 (89.7%)	1 (1.8%)	39 (100.0%)	1.90	0.43

ASCUS, atypical squamous cells of undetermined significance; CIN, Cervical Intraepithelial Neoplasia; HSIL, high-grade squamous intraepithelial lesion; ICC, invasive cervical carcinoma; LSIL, low-grade squamous intraepithelial lesion. “Inadequate sample” indicates cytology specimens insufficient for evaluation. Odds ratios for HSIL/ICC risk were calculated using logistic regression; HPV-6, HPV-11, and HPV-53 were included as low-risk or possibly oncogenic types but did not yield calculable OR values due to absence of HSIL/ICC cases. Group 1: Including the oncogenic viruses. Group 2A: Including the probably oncogenic viruses. Group 2B: Including the possibly oncogenic viruses. The *p*-value was obtained using the chi-square test.

When cytology results were evaluated, HPV positivity was below 10% in the normal cytology group, whereas it was significantly higher in ASCUS and LSIL cases (*p* < 0.001). In particular, HPV positivity exceeded 70% in HSIL cases, with HPV-16 being the predominant type. Overall, while the majority of women with normal cytology were HPV negative, the presence of cytological abnormalities was, as expected, associated with a marked increase in HPV positivity.

## 4 Discussion

In this study, we evaluated HPV prevalence, genotype distribution, and their relationship with cytological outcomes in a large cohort of 4,648 women from western Türkiye. HPV positivity was 17.7%, which is slightly lower than the prevalence reported in some regional Turkish studies, where positivity ranged between 20–25% (18). This difference may be attributed to population characteristics, screening practices, and methodological variations. Our findings showed that HPV positivity was not evenly distributed across age groups, with peaks in women younger than 30 years and in the 40–49 age group. This bimodal pattern is consistent with international observations, where an early peak corresponds to the onset of sexual activity and a later peak is thought to be related to immune senescence and viral reactivation (19, 20). Another important finding of our study was the higher frequency of multiple infections among younger women (<30 years). Although multiple infections are generally considered common in sexually active women, their clinical significance remains debated. Some studies reported no significant association between multiple infections and cytological abnormalities (21), whereas others suggested that coinfection may contribute to persistent high-risk HPV infections (20, 22). In our analysis, multiple infections were significantly more common in the younger age group, suggesting that host and behavioral factors may influence viral co-infections. Importantly, HPV-16 emerged as the dominant driver of high-grade lesions in our cohort. These results underscore the predominant influence of HPV-16 in the development of high-grade cervical lesions, aligning closely with global evidence. The lack of significant risk associated with other genotypes suggests that their oncogenic potential is comparatively limited in this population. Moreover, the finding that multiple infections do not increase HSIL risk further highlights the central role of HPV-16 rather than the overall burden of coinfection. Taken together, these findings emphasize the importance of genotype-specific surveillance and reinforce prevention strategies that particularly target HPV-16.

Genotype distribution in our cohort was dominated by HPV-16 (23%), followed by HPV-6, HPV-51, and HPV-59. This pattern is consistent with both national and international reports, which consistently show HPV-16 as the leading genotype associated with cervical cancer precursors (23, 24). The strong association between HPV-16 and high-grade lesions (HSIL, ICC) observed in this study further supports its well-established carcinogenic potential. Cytological analysis revealed that HPV positivity was below 10% in women with normal smears but exceeded 70% in those with HSIL, highlighting the strong causal relationship between persistent high-risk HPV infection and cervical carcinogenesis. Similar to our results, studies from Türkiye and Spain reported that HPV-16 was disproportionately represented in women with high-grade cytological abnormalities.

The impact of HPV vaccination on genotype distribution has been clearly demonstrated in countries with well-established immunization programs. Recent epidemiological evidence indicates that the prevalence of high-risk genotypes—particularly HPV-16 and HPV-18—declines substantially within a relatively short period following the implementation of widespread vaccination (12). This underscores the potential long-term benefits of strengthening national vaccination programs in Türkiye. The age-related bimodal distribution observed in our population is of particular clinical interest. The first peak, occurring in women younger than 25 years, aligns with findings from global meta-analyses that attribute this rise to the onset of sexual activity and increased exposure to HPV (23). The second peak in the 55–64 age group may be explained by immunosenescence, reactivation of latent infections, and hormonal changes in the peri- and postmenopausal period (19). These findings highlight the continuing need for screening not only in young women but also in older age groups who may otherwise be overlooked.

The role of multiple infections in cervical carcinogenesis remains controversial, but several recent studies suggest that coinfections may enhance viral persistence and disease progression. Luo et al. demonstrated that women with multiple infections had a significantly higher risk of persistent high-risk HPV positivity (25). Similarly, Chaturvedi et al. reported that the presence of multiple genotypes could increase the likelihood of developing cervical neoplasia. Although our data did not show increased HSIL risk in women with multiple infections, the higher rate of coinfection among younger women suggests that these infections may act as potential cofactors in a subset of individuals (26). Finally, the public health implications of our study are significant. While HPV-16 continues to be the predominant genotype associated with high-grade lesions, the detection of other high-risk types such as HPV-51 and HPV-59 at relatively higher rates underscores the potential benefits of nonavalent vaccines, which provide broader coverage. Incorporating such vaccines into the national immunization program could further reduce the burden of cervical cancer in Türkiye. Enhancing awareness campaigns and improving access to vaccination and regular screening remain essential components of effective cervical cancer prevention strategies.

### 4.1 Limitations

Several limitations should be acknowledged. The main limitation of this study is its single-center, retrospective design, which may restrict the generalizability of findings to the wider population. Another limitation is the lack of histopathological confirmation for all cytological diagnoses, which could lead to under- or overestimation of lesion severity. In addition, behavioral risk factors, prior HPV exposure, previous HPV test results, and vaccination status were not available, preventing adjustment for potential confounders. Therefore, the potential relationship between current HPV positivity and past infection or immunization could not be assessed.

### 4.2 Clinical implications

Our findings highlight the crucial role of HPV-16 in the development of high-grade cervical lesions and its disproportionate prevalence in younger women. This underlines the importance of HPV vaccination programs targeting adolescents before sexual debut. Moreover, the identification of regional genotype distribution can contribute to tailored screening strategies and inform decisions about the potential introduction of extended-valency HPV vaccines in Türkiye.

## 5 Conclusion

In this large cohort of women from western Türkiye, HPV prevalence was 17.7%, with HPV-16 emerging as the predominant genotype and showing a strong association with high-grade cervical lesions. HPV positivity demonstrated a bimodal age distribution, and multiple infections were more common in younger women, highlighting the influence of both age and infection patterns on disease dynamics. These findings emphasize the need for continued surveillance of genotype distribution, sustained cervical cancer screening across all age groups, and the implementation of effective vaccination programs tailored to regional epidemiology. Despite being largely preventable, cervical cancer remains a non-eradicated malignancy due to insufficient implementation of HPV vaccination programs worldwide.

## Data Availability

The raw data supporting the conclusions of this article will be made available by the authors, without undue reservation.

## References

[1] Bruni L Albero G Rowley J Alemany L Arbyn M Giuliano AR Global and regional estimates of genital human papillomavirus prevalence among men: a systematic review and meta-analysis. Lancet Glob Health. (2023) 11(9):e1345–62. 10.1016/S2214-109X(23)00305-4 37591583 PMC10447222

[2] World Health Organization (WHO). Human papilloma virus (HPV) and cancer. Fact Sheet (5 March 2024). Geneva: World Health Organization (2024). Available online at: https://www.who.int/news-room/fact-sheets/detail/human-papilloma-virus-and-cancer (Accessed February 13, 2026).

[3] Hull R Mbele M Makhafola T Hicks C Wang S Reis R Cervical cancer in low and middle-income countries (review). Oncol Lett. (2020) 20(3):2058–74. 10.3892/ol.2020.11754 32782524 PMC7400218

[4] Kusakabe M Taguchi A Sone K Mori M Osuga Y. Carcinogenesis and management of human papillomavirus-associated cervical cancer. Int J Clin Oncol. (2023) 28(8):965–74. 10.1007/s10147-023-02337-7 37294390 PMC10390372

[5] Brisson M Laprise JF Chesson HW Drolet M Malagón T Boily MC Health and economic impact of switching from a 4-valent to a 9-valent HPV vaccination program in the United States. J Natl Cancer Inst. (2016) 108(1):djv282. 10.1093/jnci/djv282 26438574 PMC6745694

[6] Demirci M Guzel AD Ersahin AA Yorulmaz E Ersahin SS Borsa BA. Human papillomavirus prevalence and genotype distribution among Turkish women with or without cervical lesion. Indian J Med Microbiol. (2018) 36(4):517–21. 10.4103/ijmm.IJMM_18_232 30880699

[7] Gorur L Dolanbay M Ozturk F Canoz O Donmez-Altuntas H. High-risk human papillomavirus in Turkish patients with clinically suspicious cervical lesions analyzed by multiplex-PCR. Indian J Med Res. (2022) 156(6):786–91. 10.4103/ijmr.IJMR_2335_20 37056079 PMC10278910

[8] Agadayi E Karademir D Knowledge KS. Attitudes and behaviors of women who have or have not had human papillomavirus vaccine in Turkey about the virus and the vaccine. J Community Health. (2022) 47(4):650–7. 10.1007/s10900-022-01089-1 35476168

[9] Ozyer S Uzunlar O Ozler S Kaymak O Baser E Gungor T Awareness of Turkish female adolescents and young women about HPV and their attitudes towards HPV vaccination. Asian Pac J Cancer Prev. (2013) 14(8):4877–81. 10.7314/apjcp.2013.14.8.4877 24083762

[10] Barut MU Yildirim E Kahraman M Bozkurt M Imirzalioğlu N Kubar A Human papilloma viruses and their genotype distribution in women with high socioeconomic status in Central Anatolia, Turkey: a pilot study. Med Sci Monit. (2018) 24:58–66. 10.12659/MSM.906652 29298972 PMC5764117

[11] Melo BAC Vilar LG Oliveira NR Lima PO Pinheiro MB Domingueti CP Human papillomavirus infection and oral squamous cell carcinoma—a systematic review. Braz J Otorhinolaryngol. 2021;87(3):346–52. 10.1016/j.bjorl.2020.10.017 33339760 PMC9422740

[12] Drolet M Bénard É Pérez N Brisson M Ali H Boily MC Population-level impact and herd effects following the introduction of human papillomavirus vaccination programmes: updated systematic review and meta-analysis. Lancet. (2019) 394(10197):497–509. 10.1016/S0140-6736(19)30298-3 31255301 PMC7316527

[13] Lei J Ploner A Elfström KM Wang J Roth A Fang F HPV vaccination and the risk of invasive cervical cancer. N Engl J Med. (2020) 383(14):1340–8. 10.1056/NEJMoa1917338 32997908

[14] Arbyn M Xu L Simoens C Martin-Hirsch PP. Prophylactic vaccination against human papillomaviruses to prevent cervical cancer and its precursors. Cochrane Database Syst Rev. (2018) 5(5):CD009069. 10.1002/14651858.CD009069.pub3 29740819 PMC6494566

[15] Ronco G Dillner J Elfström KM Tunesi S Snijders PJF Arbyn M Efficacy of HPV-based screening for prevention of invasive cervical cancer: follow-up of four European randomised controlled trials. Lancet. (2014) 383(9916):524–32. 10.1016/S0140-6736(13)62218-7 24192252

[16] Elfström KM Arnheim-Dahlström L von Karsa L Dillner J. Cervical cancer screening in Europe: quality assurance and organisation of programmes. Eur J Cancer. (2015) 51(8):950–68. 10.1016/j.ejca.2015.03.008 25817010

[17] Alrajjal A Pansare V Choudhury MSR Khan MYA Shidham VB. Squamous intraepithelial lesions (SIL: LSIL, HSIL, ASCUS, ASC-H, LSIL-H) of uterine cervix and Bethesda system. Cytojournal. (2021) 18:16. 10.25259/Cytojournal_24_2021 34345247 PMC8326095

[18] Ugrakli S Ozdemir M Gulseren YD Fındık S. Prevalence and genotype distribution of high-risk human papillomavirus types compared with cervical cytology. Mediterr J Infect Microb Antimicrob. (2021) 10:27. 10.4274/mjima.galenos.2021.2021.27

[19] Clifford GM Tully S Franceschi S. Carcinogenicity of human papillomavirus (HPV) types in HIV-positive women: a meta-analysis from HPV infection to cervical cancer. Clin Infect Dis. (2017) 64(9):1228–35. 10.1093/cid/cix135 28199532 PMC5399941

[20] Pisani T Cenci M. Prevalence of multiple high risk human papilloma virus (HR-HPV) infections in cervical cancer screening in Lazio region, Italy. Cancer Diagn Progn. 2024;4(1):42–5. 10.21873/cdp.10283 38173657 PMC10758846

[21] Lenselink CH Melchers WJG Quint WGV Hoebers AMJ Hendriks JCM Massuger LFAG Sexual behaviour and HPV infections in 18 to 29 year old women in the pre-vaccine era in The Netherlands. PLoS One. (2008) 3(11):e3743. 10.1371/journal.pone.0003743 19011683 PMC2581437

[22] Feng T Yang Y. Risk factors contributing to the persistent infection of high-risk human papillomavirus (HPV). Eur J Gynaecol Oncol. (2025) 46(5):1. 10.22514/ejgo.2025.060

[23] Bruni L Diaz M Barrionuevo-Rosas L Herrero R Bray F Bosch FX Global estimates of human papillomavirus vaccination coverage by region and income level: a pooled analysis. Lancet Glob Health. (2016) 4(7):e453–63. 10.1016/S2214-109X(16)30099-7 27340003

[24] Zhang L Zhang Q Pang B Tan Z Yang D Peng J Epidemiological characteristics and risk factors of high-risk HPV infection, cervical cancer, and precancerous lesions among women in Southwestern China. Front Oncol. (2025) 15:1645748. 10.3389/fonc.2025.1645748 40994964 PMC12454022

[25] Luo Q Zeng X Luo H Pan L Huang Y Zhang H Epidemiologic characteristics of high-risk HPV and the correlation between multiple infections and cervical lesions. BMC Infect Dis. (2023) 23(1):667. 10.1186/s12879-023-08634-w 37805467 PMC10560423

[26] Chaturvedi AK Engels EA Pfeiffer RM Hernandez BY Xiao W Kim E Human papillomavirus and rising oropharyngeal cancer incidence in the United States. J Clin Oncol. (2011) 29(32):4294–301. 10.1200/JCO.2011.36.4596 21969503 PMC3221528

